# Circular RNAs: implications of signaling pathways and bioinformatics in human cancer

**DOI:** 10.20892/j.issn.2095-3941.2022.0466

**Published:** 2023-03-02

**Authors:** Fan Hu, Yin Peng, Xinmin Fan, Xiaojing Zhang, Zhe Jin

**Affiliations:** Guangdong Provincial Key Laboratory of Genome Stability and Disease Prevention and Regional Immunity and Diseases, Department of Pathology, School of Basic Medical Sciences, Medical School, Shenzhen University, Shenzhen 518060, China

**Keywords:** circRNA, cancer, signaling pathway, database, bioinformatics

## Abstract

Circular RNAs (circRNAs) form a class of endogenous single-stranded RNA transcripts that are widely expressed in eukaryotic cells. These RNAs mediate post-transcriptional control of gene expression and have multiple functions in biological processes, such as transcriptional regulation and splicing. They serve predominantly as microRNA sponges, RNA-binding proteins, and templates for translation. More importantly, circRNAs are involved in cancer progression, and may serve as promising biomarkers for tumor diagnosis and therapy. Although traditional experimental methods are usually time-consuming and laborious, substantial progress has been made in exploring potential circRNA-disease associations by using computational models, summarized signaling pathway data, and other databases. Here, we review the biological characteristics and functions of circRNAs, including their roles in cancer. Specifically, we focus on the signaling pathways associated with carcinogenesis, and the status of circRNA-associated bioinformatics databases. Finally, we explore the potential roles of circRNAs as prognostic biomarkers in cancer.

## Introduction

Circular RNAs (circRNAs) are an emerging class of endogenous RNAs abundantly expressed in eukaryotic cells. These molecules are generated from precursor mRNAs through non-canonical splicing and are widely expressed in diverse species. circRNAs include exonic circRNAs, exon-intron circRNAs (EIciRNAs), and circular intronic RNAs (ciRNAs)^1,2^. circRNAs function mainly as microRNA (miR) sponges and RNA-binding protein (RBP) scaffolds, and they encode novel proteins that regulate gene transcription or protein translation^3^. Recent studies have indicated that circRNAs are involved in miR inhibition, epithelial-mesenchymal transition (EMT), and tumorigenesis. Furthermore, circRNAs expression can be tissue specific, and evidence indicates that some circRNAs are translated^4^. In contrast to linear RNAs, circRNAs form a covalently closed loop structure without a 5′ cap or 3′ tail, and have much longer half-lives^5,6^. Advances in high-throughput sequencing technology and novel bioinformatics algorithms have facilitated the systematic detection of circRNAs, most of which are stable, abundant, and conserved, and show an incredible diversity of tissue-specific expression. Studies have indicated that circRNAs are associated with many clinical characteristics and thus may provide important guidance for the accurate diagnosis and treatment of cancer^7^.

Signaling pathways play key roles in carcinogenesis. For example, the Wnt pathway is an evolutionarily conserved pathway^8,9^ that is divided into 3 classes: Wnt/β-catenin signaling, Wnt/planar cell polarity signaling, and Wnt/Ca signaling. Wnt/β-catenin signaling plays critical roles in embryonic development, tissue renewal, and regeneration^10^, and is significantly correlated with several types of cancers, such as lung cancer^11^, gastric cancer (GC)^12^, colorectal cancer (CRC)^13^, bladder cancer^14^, glioma^15^, and chronic lymphocytic leukemia^16^. Similarly, aberrant activation of the other signaling pathways has been found to significantly correlate with various cancers. Accumulating evidence indicates that circRNAs are associated with various cancer processes, including cancer initiation, progression, and metastasis, *via* signaling pathways^17^.

Traditional experimental approaches have been important in exploring the biological functions and characteristics of molecules as well as cancer pathogenesis. However, these methods can be time-consuming and laborious. With the discovery of large numbers of circRNAs, an urgent need exists to use *in silico* methods to reveal their characteristics, and guide the rational design of expensive and laborious clinical trials^2^.

In this review, we summarize current understanding of the biological characteristics and functions of circRNAs, with a focus on signaling pathways associated with carcinogenesis. This information should provide insights into potential new targets for the treatment of cancers. Finally, we discuss the current status of circRNA bioinformatics databases, and explore the potential roles of circRNAs as prognostic biomarkers and therapeutic targets in cancer.

## RNA circularization and circRNA biogenesis

**Figure 1:**
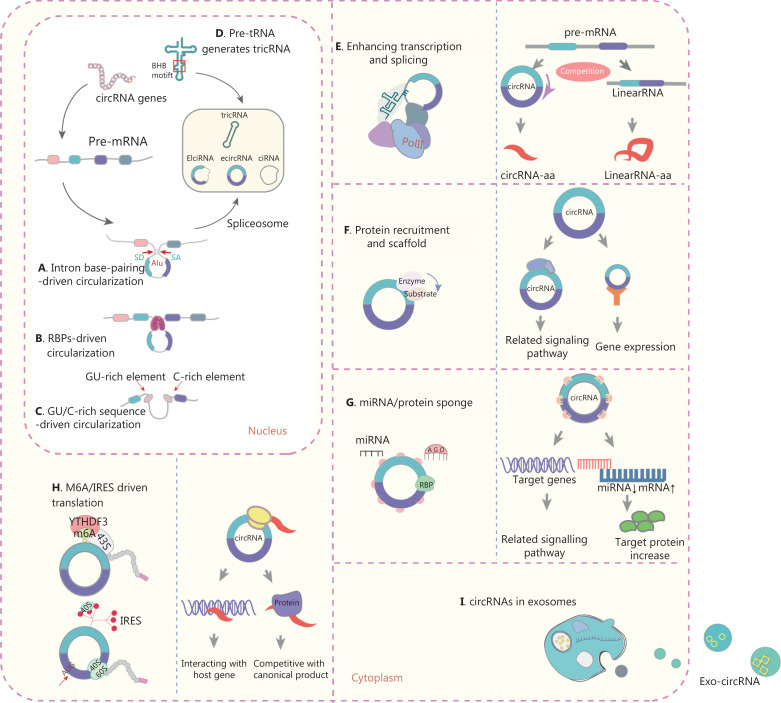
Biogenesis, mechanisms, and functions of circRNAs in cancer. (A) Intron base-pairing-driven circularization. (B) RBP-driven circularization. Looping of the introns (containing the splice donor site and splice acceptor site) flanked by exons is required for back-splicing. This looping can be facilitated by base-paring of complementary sequences between inverse-repeat *Alu* elements (A) or by RBP dimerization (B). RBPs bind intron-flanking introns and promote circularization of the pre-mRNA (or lariat), thus generating circRNAs. (C) GU/C-rich sequence-driven circularization. Pre-mRNAs comprising a 7-nucleotide (nt) GU-rich element and an 11-nt C-rich element consensus motif facilitate the generation of circRNAs. (D) Pre-tRNAs generate tricRNAs. An intron-containing pre-tRNA is cleaved by the tRNA spicing endonuclease (TSEN) complex, thus generating a tricRNA at the bulge-helix-bulge (BHB) motif; the intron termini then ligate and form a tricRNA. (E) circRNAs interact with Pol II, thereby regulating parental gene transcription and splicing. Competition between linear splicing and back-splicing of the pre-mRNA influences the balance between the 2 types of splicing. (F) circRNAs interact with proteins in several ways. circRNAs act as scaffolds that facilitate interactions between enzymes with their substrates. circRNAs can also recruit proteins to specific loci and promote protein assembly. (G) circRNAs function as miR and protein sponges. circRNAs containing miR response elements (MREs) can regulate miR-target mRNA expression through miR sequestration (or “sponging”). A highly expressed circRNA with many MREs is likely to function as an miR sponge and to positively regulate target mRNA translation. circRNAs containing binding motifs for RBPs might sponge these proteins and regulate their functions. (H) m6A and IRES-driven circRNA translation. A subset of circRNAs containing IRES and/or m6A modifications can serve as templates for translation and give rise to circRNA-specific peptides with the ORF crossing the back-splicing junctions. (I) circRNA transport *via* exosomes. circRNAs can be loaded into exosomes, thereby forming exo-circRNAs, which function as messengers in intercellular communication through the horizontal transfer of their cargo molecules to recipient cells.

circRNAs are derived from pre-messenger RNAs (pre-mRNAs) and originate from exons, introns, antisense RNAs, and intergenic regions. Under non-pathological conditions, circRNAs control gene expression by regulating gene transcription, RNA splicing, and scaffold assembly^18,19^. In addition, some circRNAs encode functional peptides. Four mechanisms of circularization have been confirmed: intron base-pairing-driven circularization (**Figure 1A**), RBP-driven circularization (**Figure 1B**), GU/C-rich sequence-driven circularization (**Figure 1C**), and pre-tRNA-mediated generation of tRNA intronic circular RNA (tricRNA) (**Figure 1D**)^2^. Recent studies have revealed that chromosomal translocations lead to the generation of fused circRNA^20^. The most common types of circRNA are ciRNAs, EIciRNAs, and exonic circRNAs, which account for the largest proportion (85%). EcircRNAs are distributed mainly in the cytoplasm, whereas ciRNAs and EIciRNAs exist mainly in the nucleus^21,22^.

In eukaryotes, pre-mRNAs are generally processed to generate linear mRNAs through canonical splicing, whereas circRNAs are formed through alternative “head-to-tail” back-splicing events^20^, in a process involving the formation of a covalently closed loop through reverse ligation of a downstream-splice donor site to an upstream-splice acceptor site. circRNA circularization is promoted by RBP-mediated bridging of relevant intronic sequences in RNA^1,20,22^. Studies on circRNA biogenesis have expanded the understanding of the complexity of RNA transcriptional regulation; however, the mechanism of the back-splicing events that generate circRNAs remains to be fully elucidated.

## Potential functions of circRNAs in cancer

### Interactions with proteins as regulators and scaffolds

#### Transcription and splicing

Some circRNAs interact with RNA polymerase II (Pol II), and consequently regulate the transcription and splicing of parental genes. In the nucleolus, EIciRNAs and ciRNAs enhance parental gene transcription by interacting with the U1 small nuclear ribonucleoprotein or binding the Pol II promoter^5^. Similarly, ci-ankrd52 and ci-sirt7 localize to and interact with the elongating Pol II complex. Depletion of these ciRNAs decreases the transcription levels of the ankyrin repeat domain 52 (ANKRD52) or sirtuin 7 (SIRT7) genes^23^. Circ-DNMT1 promotes the nuclear translation of p53 and acts on AU-rich element RNA-binding protein 1 (AUF1), thereby resulting in cellular autophagy and target Dnmt1 mRNA stability in breast cancer^24^. Thus, circRNAs compete with spliced pre-mRNAs by triggering transcription, and consequently balance the levels of circRNAs and corresponding mRNAs (**Figure 1E**).

#### Protein recruitment and scaffolding

Some circRNAs serve as scaffolds that promote protein recruitment and assembly. A recent report has demonstrated that circndufb2 functions as a scaffold that binds the IGF2BP proteins with TRIM25, a positive regulator of tumor progression and metastasis in non-small cell lung cancer (NSCLC)^25^. The circRNA FECR1, which is generated from the Friend leukemia virus integration 1 (FLI-1) oncogene, recruits TET1 to the promoter of FLI-1 and promotes breast cancer metastasis^26^. Some circRNAs also facilitate reaction kinetics by binding enzymes and substrates. Circ-Foxo3 halts cell cycle progression by forming a ternary complex with cyclin-dependent kinase 2 and its inhibitor p21^27^. Thus, circRNAs can act as scaffolds that mediate complex formation by specific enzymes and substrates involved in carcinogenesis (**Figure 1F**).

### miRs and protein sponges

circRNAs inhibit the functions of miRs by competitive binding or “sponging” through the formation of stable complementary interactions. The circRNA-miR-mRNA axis is also involved in various cancer-associated pathways with both agonistic and antagonistic effects on carcinogenesis. CIRS-7, the first circRNA identified as an miR sponge, inhibits circRNA-7, through more than 70 conventional miR-7 binding sites, and is associated with cancer progression^28,29^. Moreover, circRUNX1 promotes papillary thyroid cancer (PTC) progression and metastasis by sponging miR-296-3p and regulating DDHD2 expression^30^. The circRNA UBE2Q2 promotes malignant progression of GC by regulating the miR-370-3p/STAT3 axis^31^. CircORC5 suppresses GC progression by sponging miR-30c-2-3p and regulating AKT1S1^32^. However, the miR sponge model is becoming increasingly controversial, because most circRNAs do not show strong sponging effects on miR-binding sites^33^. Furthermore, the circRNA-miR axis regulates the activity of the corresponding linear mRNA. Thus, the mechanisms through which circRNAs act as miR sponges, and consequently regulate gene and protein expression, require further investigation. In another model, the biological function of circRNAs depends on interactions with RBPs, which have various roles in circRNA splicing, processing, folding, stabilization, and localization^4,34^. RBPs interact with circRNAs and form RNA-protein complexes, which in turn regulate the circularization of circRNAs, such as SCD-circRNA and circPCNX, in cancers^35,36^. RBPs also interact with circRNAs, and hide or expose certain regions in a process essential for the correct splicing, localization, and translation of cellular components^34^. Thus, sponging of circRNAs by miRs or proteins is a key mechanism through which circRNAs perform multiple functions in cancer (**Figure 1G**).

### circRNA translation

circRNAs initially lack a 5′-cap and 3′-tail, and are therefore classified as non-coding RNAs; however, a small fraction of circRNAs (< 1%) are translated into functional proteins or micropeptides *via* one of 2 mechanisms: N6-methyladenosine (m6A)-driven or internal ribosome entry site (IRES)-driven translation. The m6A motif in the 5′-untranslated region (5′-UTR) is a major mechanism^37–39^ (**Figure 1H**). Yang et al.^39^ have shown that m6A directly recruits the initiation factor eIF4G2 for formation of the 43S complex, which in turn promotes the initiation of circRNA translation in human cells. IRES elements within the 5′-UTRs of the upstream open reading frames (ORFs) function as RNA regulatory elements that initiate circRNA translation independently of the 5′ cap structure^40–48^. Xia et al.^41^ have suggested that circ-AKT3 is generated by the circularization of exons 3–7 of AKT3, which contain both an ORF and an IRES sequence, and have speculated that the 5′-UTR of circ-AKT3 is structurally folded into the IRES and encodes the AKT3-174 aa protein. Interestingly, circ-EGFR forms a polymetric novel protein complex known as rolling-translated EGFR^48^. We believe that this model provides a new understanding of circRNAs that blurs the definition of non-coding RNAs. Thus, circRNA-encoded peptides stand to become a new resource for anti-tumor protein drug screening of early tumor biomarkers, precise therapeutics, and molecules to aid in prognostication.

### Exosome-circRNAs (exo-circRNAs) in cancer

Exosomes are small extracellular vesicles (EVs) of endocytic origin that are secreted by most cell types. During carcinogenesis, exosomes function as messengers for intercellular communication. Moreover, circRNAs can be loaded into exosomes, thus forming exo-circRNAs, which communicate with neighboring or distant cells through horizontal transfer of their cargo molecules to recipient cells^49^ (**Figure 1I**). Exo-circRNAs influence cancer progression and metastasis by altering biological signaling pathways. A recent report has indicated that circLPAR1 is encapsulated in exosomes and is diminished in CRC tissues. Furthermore, in plasma exosomes, circLPAR1 expression is markedly downregulated in CRC development but recovers after surgery^50^. Currently, 2 hypotheses may explain the function of exo-circRNAs: cell communication and circRNA clearance^51,52^. Huang et al.^51^ have reported that exosomal circRNA-100338 enhances the metastatic ability of hepatocellular carcinoma (HCC) cells by transferring circRNA-100338 encapsulated in EVs to recipients. Alternatively, Lasda and Parker^52^ have hypothesized that exosomes eliminate endogenous cellular circRNAs *via* extracellular vesicles, because circRNAs are more enriched than linear forms in EVs. However, Alhasan et al.^53^ have proposed that circRNA enrichment in exosomes results from the presence of exosomal exonucleases. Regardless of the mechanism, exosomes are readily accessible and protect RNAs from degradation in various human biofluids. Together, these findings implicate exo-circRNAs as valuable biomarkers and therapeutic targets in human diseases.

## Multiple signaling pathways of circRNAs in cancers

Multiple signaling pathways are closely associated with carcinogenesis. The complexity of signals and their functional roles are crucial for the development and growth of cancers, as well as other diseases. Signaling pathway dysregulation has been recognized in a variety of human cancers^8,54–56^. In addition, some signaling pathways induce EMT, a crucial driver of cancer progression. During this process, which is considered a trigger of cancer metastasis, epithelial cells lose their polarity and differentiated state, and acquire a mesenchymal-like phenotype. Some circRNAs have been found to act as competing endogenous RNAs (ceRNAs) for miRs involved in EMT signaling pathways, thus leading to tumor progression^57^, although the underlying mechanisms are uncertain. In this review, we dissect the roles of signaling pathways in malignant carcinomas, focusing on molecular mechanisms and prospects for future intervention (**Table 1**).

**Table 1:** Overview of circRNA functions and mechanisms in multiple signaling pathways

PI3K/AKT/mTOR signaling pathway							
Pathway	Cancer	CircBase name	Location	Regulation	Mechanism		Putative function
PI3K/AKT/mTOR	CRC	circCDYL	C	Down	miR sponge/ceRNA	circCDYL/miR-150-5p promotes apoptosis	Represses cellular growth and migration
		circ_0001313	NI	Up		circ-0001313/miR-510-5p/AKT2 axis	Promotes development and progression of cancer
		hsa_circ_002144	C	Up		hsa_circ_002144/miR-615-5p/LARP1/mTOR axis	Promotes growth and metastasis
		circIL4R	C	Up		circIL4R/miR-761/TRIM29/PHLPP1	Promotes proliferation and metastasis
	GC	circNRIP1	C	Up		circNRIP1/miR-149-5p/AKT1/mTOR	Promotes GC proliferation, migration, and invasion
	Glioma	circ_0014359	C	Up		circ_0014359/miR-153/PI3K	Promotes glioma progression
	NSCLC	circFGFR3	NI	Up		circFGFR3/miR-22-3p/Galectin-1-AKT/ERK1/2 signaling	Galectin-1-AKT/ERK1/2 signaling
	HCC	circRNA-100338	C	Up		circRNA-100338/miR-141-3p/RHEB/mTOR	Poor prognosis
	Ovarian cancer	circPLEKHM3	C	Down		circPLEKHM3/miR-9/BRCA1/DNAJB6/KLF4/AKT1 axis	Inhibits cell growth, migration, and EMT
	Breast cancer	circKDM4B	C	Down		circKDM4B/miR-675/NEDD4L	Inhibits cell angiogenesis and tumor metastasis
	PTC	circ_0067934	NI	Up		circ_0067934/miR-1301-3p/PI3K/AKT signaling pathway	Promotes cancer progression
	Pancreatic cancer	circ-ANAPC7	C	Down		circ-ANAPC7/miR-373/PHLPP2	Inhibits muscle wasting and cancer cachexia
	CRC	circ_LNLM	C	Up	Scaffold/binding with AKT	Blocks ubiquitination of AKT	Promotes the early metastasis especially for lymph node-negative CRC patients with synchronous liver metastasis
	OSCC	circ_0007059	NI	Down	Epigenetic modification	AKT/mTOR signaling pathway	Suppresses cell growth, migration, and invasion; facilitates apoptosis
	Glioblastoma	circ-AKT3	C	Down	Translation	AKT3-174 aa competitively interacts with phosphorylated PDK1	Inhibits glioblastoma tumorigenicity

Wnt/β-catenin signaling pathway							
Pathway	Cancer	CircBase name	Location	Regulation	Mechanism		Putative function
Wnt/β-catenin	CRC	hsa_circ_0000523	NI	Down	miR sponge/ceRNA	hsa_circ_0000523Wnt/β-catenin	Inhibits cancer progression
		hsa_circ_0009361	C	Down		hsa_circ_0009361/miR-582/APC2 network	Induces cancer progression and EMT
		circRNA_100290	NI	Up		circRNA_100290/miR-516b/FZD4/Wnt/β-catenin	Oncogenic function
	DLBL	circEAF2	NI	Up		miR-BART19-3p/APC/β-catenin axis	Oncogenic function
	EC	hsa_circ_0002577	NI	Up		miR-197/CTNND1 axis, Wnt/β-catenin pathway	Oncogenic function
	Glioma	hsa_circ_0000177	NI	Up		hsa_circ_0000177/miR-638/FZD7-Wnt	Promotes cancer proliferation and invasion
	Bladder cancer	circPRMT5	C	Up		circPRMT5/miR-30c/Snail/E-cadherin	EMT and cancer metastasis
	Lung cancer	circ_0007059	NI	Down		circ_0007059/miR-378/Wnt/β-catenin	Inhibits progression and EMT process
	GC	circAXIN1	C	Up	Translation	Competitively interacts with APC	AXIN1-295 aa promotes GC progression
	TNBC	circ-EIF6	C	Up		circ-EIF6/EIF6-224 aa/MYH9/Wnt/β-catenin	Promotes TNBC progression
	Liver cancer	circβ-catenin	C	Up		Stabilizes full-length β-catenin by antagonizing degradation	Promotes liver cancer cell growth
	Colon cancer	circFNDC3B	C	Down		circFNDC3B-218 aa/Snail/FBP1	Inhibits cell proliferation, invasion, migration, and EMT
	RCC	circESRP1	C	Down	Feedback loop	circESRP1/miR-3942/CTCF/c-Myc/EMT	Inhibits cell proliferation and EMT
	HCC	circ-ZNF652	C	Up		circ-ZNF652/miR-203/Snail feedback loop	EMT and cancer metastasis
						circ-ZNF652/miR-502-5p/Snail feedback loop	EMT and carcinoma metastasis
	PTC	circRNA_102171	N	Up	TF	Modulates CTNNBIP1-dependent activation of the β-catenin pathway	Promotes PTC progression
	LSCC	circMTCL1	C	Up		Inhibits C1QBP ubiquitin degradation	Promotes progression

Notch and Hippo signaling pathways							
Pathway	Cancer	CircBase name	Location	Regulation	Mechanism		Putative function
Notch	Glioma	circNFIX	NI	Up	miR sponge/ceRNA	circNFIX/miR-34a-5p/NOTCH1 pathway	Oncogenic function
	LADC	circ-MTO1	NI	Down		circ-MTO1/miR-17/QKI-5 feedback loop	Negatively regulates LUAD
	Breast cancer	circFAT1	NI	Up		circFAT1/miR-525-5p/spindle	Promotes cancer cell metastasis
	PDAC	circ-ASH2L	C	Up		circ-ASH2L/miR-34a	Oncogenic function
	CRC	circAPLP2	NI	Up		circAPLP2/miR-101-3p inhibits apoptosis	Promotes proliferation and metastasis
	T-ALL	circPVT1	NI	Up		circPVT1/miR-30e/DLL4	Promotes cell proliferation
	RCC	circPDK1	C	Up		circPDK1/miR-377-3P-NOTCH1	Promotes RCC cell migration and invasion
Hippo	GC	circRNA-000425	NI	Down	miR sponge/ceRNA	YAP1/circRNA-000425/miR-17 and miR-106	Inhibits cancer cell growth
	HCC	circ_104075	NI	Up		circ_104075/miR-582-3p/YAP1	Oncogenic function
	Osteosarcoma	circPIP5K1A	NI	Up		circPIP5K1A/miR-515-5p/YAP axis	Promotes cancer cell invasion and migration, and cancer stemness
	ICC	circACTN4	N and C	Up		circACTN4/miR-424-5p/YBX1/FZD7	Promotes tumor progression
	CRC	circ-LECRC	C	Down	Feedback loop	circ-LECRC/miR-135b-5p/KLF4; suppresses activation of YAP signaling	Inhibits tumor growth
	Colon cancer	circPPP1R12A	C	Up	Translation	circPPP1R12A-73 aa/Hippo-YAP	Promotes tumor pathogenesis and metastasis
	CRC	circ1662	C	Up	Nuclear accumulation	Promotes YAP1 nuclear accumulation and regulates smad3	Promotes CRC cell invasion and migration
	Breast cancer	circYap	NI	Down		circYap/eIF4G/PABP; negatively regulates YAP translation	Inhibits tumor growth

p53/Bcl2 signaling pathway							
Pathway	Cancer	CircBase name	Location	Regulation	Mechanism		Putative function
p53/Bcl2	NSLC	circVANGL1	NI	Up	miR sponge/ceRNA	circVANGL1/miR-195/Bcl-2 axis	Inhibits proliferation and induced apoptosis
	HCC	circ-BIRC6	C	Up		circ-BIRC6/miR-3918/Bcl2 axis	Oncogenic function
	CRC	circ_0021977	NI	Down		circ_0021977/miR-10b-5p/p21/p53 axis	Suppresses cancer proliferation, migration, and invasion
	HNSCC	circPVT1	NI	Up		circPVT1/miR-497-5p/mut-p53/YAP/TEAD complex	Oncogenic function
	NSCLC	hsa_circ_0002874	NI	Up		hsa_circ_0002874/miR-1273f/MDM2/p53	Promotes resistance of NSCLC
	GC	hsa_circ_006100	NI	Up		hsa_circ_006100/miR-195/GPRC5A/Bcl-2 axis	Oncogenic function
	Osteosarcoma	circ_0001785	NI	Up		circ_0001785/miR-1200/HOXB2/Bcl-2 family	Oncogenic function
	OSCC	hsa_circ_0055538	NI	Down	Apoptosis	p53/Bcl-2/caspase signaling pathway	Inhibits tumor growth
	Osteosarcoma	circ_0007534	NI	Up		Bcl-2/caspase-3, AKT/GSK-3β pathway	Regulates cancer growth and apoptosis
	CRC	circZNF609	C	Down		circZNF609/p53	Promotes cancer apoptosis
	Glioma	circCHAF1A	NI	Up	Feedback loop	circCHAF1A/miR-211-5p/HOXC8/MDM2-p53	Facilitates glioma proliferation and tumorigenesis
		CDR1as	N	Down	Disruption of the p53/MDM2 complex	CDR1as directly interacts with p53	Inhibits gliomagenesis
	Breast cancer	circ-Ccnb1	NI	Down	mut-p53	circ-Ccnb1/H2AX/Bclaf1 complex inhibits mut-p53	Inhibits breast cancer progression
		circ-DNMT1	NI	Up	p53 nuclear translocation	Binding to p53 promotes nuclear translocation of both p53 and AUF1	Enhances breast cancer progression

TGF-β/Smad signaling pathway inducing EMT							
Pathway	Cancer	CircBase name	Location	Regulation	Mechanism		Putative function
TGF-β/Smad	Bladder cancer	circRIP2	C	Down	miR sponge/ceRNA	circRIP2/miR-1305/TGF-β2/smad3	EMT and cancer progression
	NSCLC	circPTK2	C	Down		circPTK2/miR-429/miR-200b-3p/TGF-β/Smad	Inhibits EMT and metastasis
	OSCC	circANKS1B	C	Up		circANKS1B/miR-515-5p	Affects metastatic potential, EMT, and cisplatin resistance
	TNBC	circANKS1B	C	Up		circANKS1B/miR-148a-3p and miR-152-3pUSF1/TGF-β1/Smad	EMT and metastasis
	ESCC	circ-DOCK5	C	Down	Feedback loop	ZEB1/circ-DOCK5/miR-627-3p/TGF-β/Smad	EMT and metastasis
	OSCC	circUHRF1	C	Up		circUHRF1/miR-526b-5p/c-Myc/TGF-β1/ESRP1 feedback loop	Accelerates EMT and tumorigenesis
	CRC	circPTEN1	C and N	Down		Decreases nuclear translocation of Smad complexes	Suppresses cancer progression and EMT

C, cytoplasm; N, nucleus; NI, not investigated.

### circRNAs and the PI3K/AKT/mTOR signaling pathway

**Figure 2:**
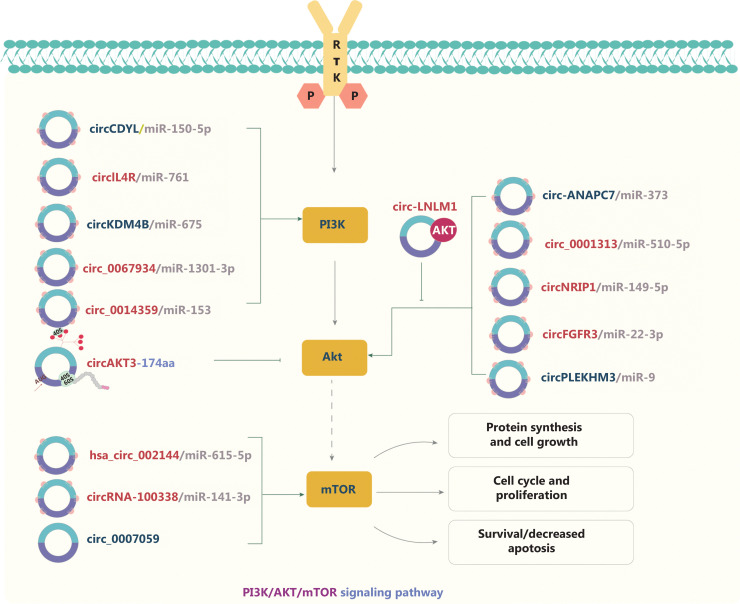
The molecular roles of circRNAs in the PI3K/AKT/mTOR signaling pathway. The PI3K/AKT/mTOR pathway is the most commonly activated pathway in human cancers. Receptor tyrosine kinase activation and tyrosine phosphorylation of its cytosolic domain or its scaffolding adaptors create binding sites that recruit the lipid kinase PI3K—a regulator of signaling and intracellular vesicular trafficking and cellular processes such as proliferation, survival, and protein synthesis—to the plasma membrane. circRNAs commonly act as ceRNAs of miRs in tumor progression, thus inhibiting the functions of miRs. Nine upregulated and 6 downregulated circRNAs are indicated; 12 circRNAs regulate the PI3K/AKT/mTOR pathway through ceRNA effects. Red: upregulated circRNAs; blue: downregulated circRNAs.

The PI3K/AKT/mTOR signaling pathway is highly activated in various cancers, and is mediated by upstream oncoproteins receptor tyrosine kinases, *RAS* oncogenes, or G-protein-coupled receptors (**Figure 2**). Dysregulation of the PI3K/AKT/mTOR signaling pathway is associated with tumorigenesis. Therefore, studies of the interactions between circRNAs and the PI3K/AKT signaling pathway have become a major research focus^58,59^. circRNAs commonly act as ceRNAs for miRs in tumor progression. On the basis of the ceRNA mechanism, downstream pathways are activated or repressed by sponging miRs. In CRC, for example, circCDYL^58^, circ_0001313^59^, hsa_circRNA_002144^60^, and circIL4R^61^ regulate the PI3K/AKT signaling pathway *via* the ceRNA mechanism, thereby promoting or inhibiting tumor progression. CircNRIP1 serves as an miR-149-5p sponge that promotes GC progression *via* the AKT1/mTOR pathway^62^. In NSCLC, circFGFR3 regulates both the AKT and ERK1/2 signaling pathways by sponging miR-22-3p^63^. Elevated circRNA-100338 activates the mTOR signaling pathway in HCC *via* the circRNA-100338/miR-141-3p/*RHEB* axis and is associated with poor prognosis in patients with hepatitis B-associated HCC^64^. Shi et al.^65^ have indicated that circ_0014359 sponges miR-153, and consequently regulates p-AKTser473 expression and accelerates glioma progression. CircPLEKHM3^66^ and circKDM4B^67^ also sponge miRs, thus promoting or inhibiting the AKT signaling pathway in ovarian cancer and breast cancer, respectively. Moreover, circRNAs have been found to modulate tumor progression *via* epigenetic modification events. For example, circ-0124554 (circ-LNLM) promotes CRC hepatic metastasis by blocking AKT ubiquitination^68^. In addition, circ_0067934 and circ_0007059 influence malignant cell behavior by phosphorylating AKT/mTOR in oral squamous cell carcinoma (OSCC) and thyroid carcinoma, respectively^69,70^. A recent study has indicated that circ-ANAPC7 regulates the CREB-miR-373-PHLPP2 feed-forward loop *via* the PHLPP2-AKT-TGF-β signaling axis, thus inhibiting tumor growth and muscle wasting in pancreatic cancer^71^. Interestingly, circRNAs encoding a novel protein have been found to affect tumor development *via* the PI3K/AKT/mTOR signaling pathway. For instance, a tumor suppressor protein encoded by circ-AKT3 RNA inhibits glioblastoma tumorigenicity by competing with active phosphoinositide-dependent kinase-1^41^. These findings indicate that circRNAs not only function as oncogenic promoters but also participate in protein modification.

### circRNAs and the Wnt signaling pathway

**Figure 3:**
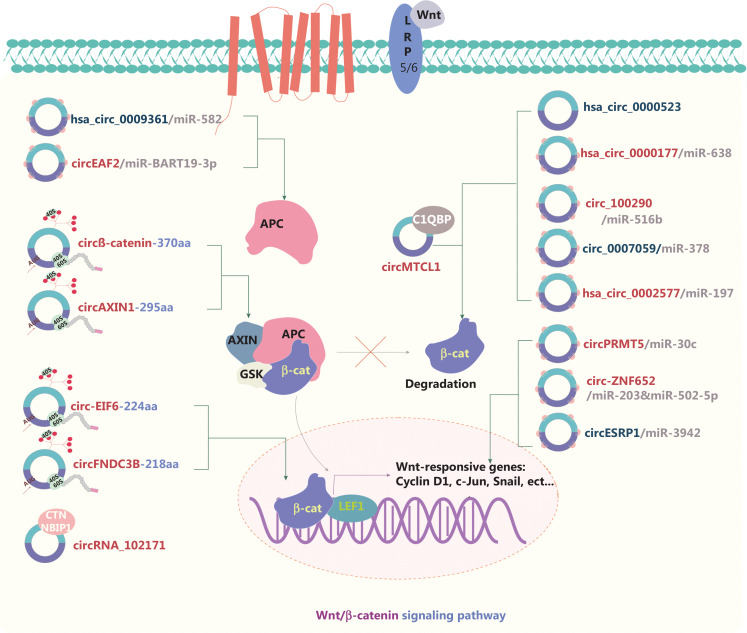
Molecular roles of circRNAs in the Wnt/β-catenin signaling pathway. The Wnt pathway is critical for various cellular functions, such as ensuring cell polarity, movement, and proliferation; this pathway is also often involved in cancer progression. A destruction complex initially phosphorylates and then ubiquitinates β-catenin in the cytoplasm, and subsequently inhibits Wnt signaling. circRNAs regulate the β-catenin destruction complex, thereby activating or suppressing the nuclear downstream targets of Wnt signaling. circRNA competition with the endogenous RNAs (ceRNAs) of miRs is their main mechanism of regulating the Wnt signaling pathway. The IRES-driven translation of novel proteins from circRNAs is another crucial mechanism of circRNAs in Wnt signaling. Eleven upregulated and 5 downregulated circRNAs are shown; 8 of these circRNAs regulate the Wnt pathway through ceRNA effects. Red: upregulated circRNAs; blue: downregulated circRNAs; β-cat: β-catenin.

The activity of the canonical Wnt signaling pathway is positively associated with carcinogenesis. In brief, β-catenin, axin, glycogen synthase kinase-3 (GSK-3), adenomatous polyposis coli (APC), and casein kinase 1 form a destruction complex that initially phosphorylates and subsequently ubiquitinates β-catenin in the cytoplasm (**Figure 3**). The formation of this complex is blocked by the presence of Wnt ligands. Consequently, the main protein β-catenin is transferred from the extracellular environment to the nucleus, where it activates Wnt-targeted downstream proteins^72–74^. On the basis of the ceRNA mechanism, downstream pathways are activated or repressed by sponging of miRs^75–77^. In the cytoplasm, circRNAs affect Wnt activation by interacting with proteins within the destruction complex. APC, a key protein in the destruction complex, negatively regulates the Wnt/β-catenin signaling pathway. Geng et al.^72^ have revealed the tumor suppressive function of hsa_circ_0009361 as well as its ability to sponge miR-582 and consequently increase the expression of APC2, which in turn affects Wnt/β-catenin signaling in CRC. In the Wnt signaling pathway, Wnt ligands bind Frizzled (FZD) receptors, the lipoprotein-related protein (LRP) or the Wnt antagonist Dickkopf-1 (Dkk1) at the cell surface, thus activating or suppressing downstream pathways^78^. DKK1 specifically binds LRP5/6, thereby interfering with formation of the Wnt-LRP5/6-FZD complex and inhibiting the downstream Wnt signaling pathway. In CRC and glioma, circ_100290, hsa_circ_0000177, and hsa_circ_0000523 regulate these surface proteins and are involved in the Wnt pathway^13,15,79^.

When β-catenin enters the nucleus, some circRNAs interact with the genes downstream of the Wnt pathway. CircMTCL1 promotes advanced laryngeal squamous cell carcinoma progression by inhibiting C1QBP ubiquitin degradation and mediating β-catenin accumulation in the cytoplasm and nucleus^80^. In endometrial carcinoma, the hsa_circ_0002577/miR-197/CTNND1 axis affects the expression of β-catenin, cyclin D1, and c-Myc, thus activating the Wnt signaling pathway^81^. In PTC, Bi et al.^82^ have demonstrated that circRNA_102171 interacts with CTNNBIP1 and subsequently blocks its interaction with the β-catenin/TCF3/TCF4/LEF1 complex, thus activating the Wnt/β-catenin signaling pathway. Some circRNAs encode novel proteins that affect the Wnt pathway^12,74,83,84^. Our group has demonstrated that the AXIN1-295 aa protein encoded by circAXIN1 competitively interacts with APC, thus leading to dysfunction of the “destruction complex” in GC^12^. In HCC, circβ-catenin encodes a novel protein, circβ-catenin-370 aa, which stabilizes β-catenin and leads to activation of the Wnt signaling pathway^74^. Moreover, hsa_circ_0007059 appears to hinder the interaction between Wnt3a and β-catenin, and consequently inhibit EMT in lung cancer^85^. circRNAs have also been shown to form a feedback loop that regulates cancer development. Guo et al.^76^ have reported that circ-ZNF652 interacts with miR-203 and miR-502-5p, which target Snail, thus promoting metastasis in HCC. In turn, Snail upregulation increases circ-ZNF652 expression by binding its promoter, thereby forming a positive feedback loop in HCC. CircESRP forms a positive feedback loop regulating cancer progression *via* EMT^86^. Thus, studies have confirmed the critical roles of circRNAs in the circRNA/Wnt/β-catenin signaling pathway; if these findings are further validated, they may have potential novel therapeutic applications for cancer.

### circRNAs and the Notch and Hippo signaling pathways

**Figure 4:**
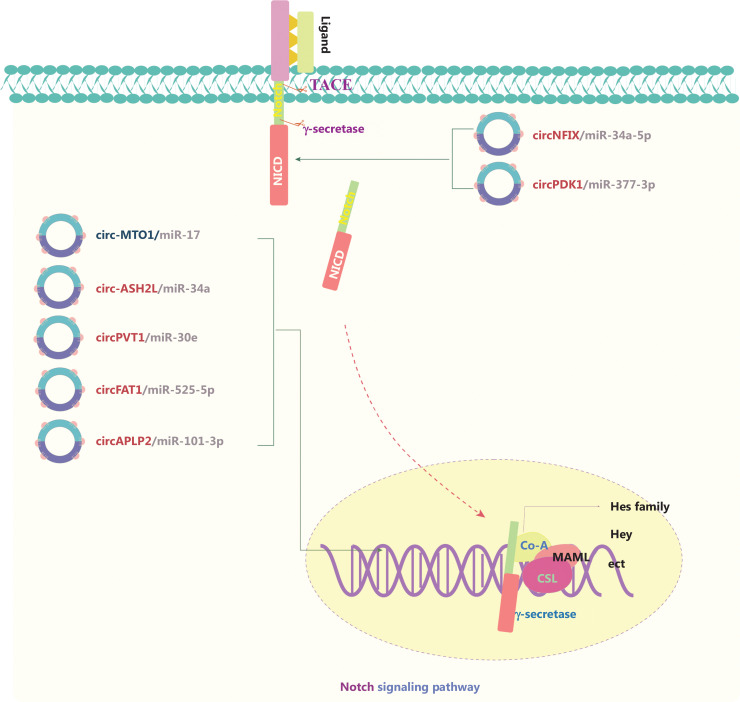
The molecular roles of circRNAs in the Notch signaling pathway. The Notch pathway is responsible for neurogenesis, angiogenesis, and cell proliferation, and it directly couples events at the cell membrane with the regulation of transcription. Notch receptors bind specific ligands, thus resulting in sequential cleavage of the Notch receptor and the release of the Notch intracellular domain (NICD) into the signal-receiving cell. The NICD containing the nuclear localization sequence translocates to the nucleus. The NICD interacts with the CBF-1/Su(H)/LAG1 (CSL) transcription factor and subsequently induces the recruitment of the transcriptional co-activator (Co-A) Mastermind-like (MAML) and other transcriptional Co-As. All 4 receptors (Notch1–4) mediate canonical signaling by activating CSL-dependent transcription and are involved in cancers. Six upregulated and one downregulated circRNAs are shown; 7 circRNAs regulate the Notch pathway through ceRNA effects. Red: upregulated circRNAs; blue: downregulated circRNAs.

The Notch signaling pathway is responsible for neurogenesis, angiogenesis, and overall cell survival and proliferation. Notch receptors (NOTCH1, NOTCH2, NOTCH3, and NOTCH4) are transmembrane proteins that bind specific ligands, thus resulting in the activation of a series of biochemical events (**Figure 4**)^87^. circRNAs in Notch signaling generally serve as ceRNAs that sponge miRs, and activate pathways through directly affecting receptors and/or its ligands^88–94^. Abnormal Notch signaling is usually associated with genetic mutations of crucial factors, particularly NOTCH1. Xu et al.^88^ have found that circNFIX sponges miR-34a-5p and targets NOTCH1, thereby activating the Notch signaling pathway in glioma. Circ-MTO1 has also been identified as a tumor suppressor that functions as part of the circ-MTO1/miR-17/QKI-5 feedback loop in inhibiting lung adenocarcinoma progression by inactivating the Notch signaling pathway^89^. Circ-ASH2L promotes tumor progression by sponging miR-34a and consequently regulating Notch1 in pancreatic ductal adenocarcinoma^91^. On the basis of these findings, circRNAs involved in Notch signaling have been implicated as a novel strategy to prevent cancer progression.

**Figure 5:**
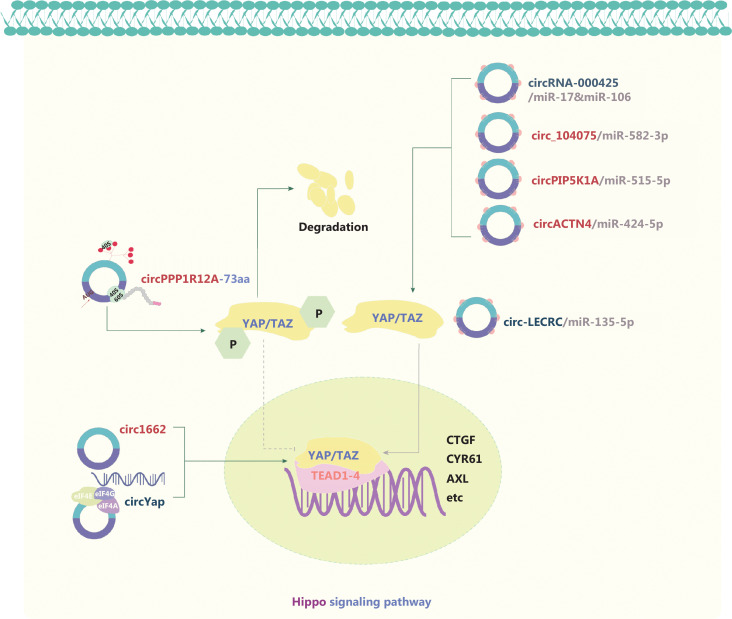
Molecular roles of circRNAs in the Hippo signaling pathway. The Hippo pathway is an evolutionarily conserved signaling pathway with key roles in various diseases, most notably cancer. The Hippo kinase cascade converges on its nuclear effector YAP/TAZ and regulates gene expression programs. YAP/TAZ phosphorylation by Hippo signaling inactivates YAP/TAZ transcriptional coactivators by excluding them from the nucleus and promotes YAP/TAZ degradation. When Hippo signaling is low, YAP/TAZ enters the nucleus, where it drives gene expression. Five upregulated and 3 downregulated circRNAs are indicated; 4 circRNAs regulate the Hippo pathway through ceRNA effects. Red: upregulated circRNAs; blue: downregulated circRNAs.

The Hippo signaling pathway comprises several tumor-suppressors and oncogenes. When the Hippo signaling pathway is inactivated, the Yes-associated protein (YAP) and PDZ-binding motif are activated and translocate into the nucleus, where they promote cell proliferation^95–97^ (**Figure 5**). Various studies have shown the involvement of circRNAs in the Hippo signaling pathway, with dominant mechanisms including ceRNA competition^98–100^, novel protein translation^96^, a feedback loop^101^, and nuclear accumulation of YAP^102^. YAP derived circ-LECRC functions as a “brake signal” that suppresses hyperactivation of oncogenic YAP signaling in CRC^101^. Louis and Coulouarn^54^ have found that circACTN4 upregulates YAP1 expression by sponging miR-424-5p, and recruits Y-box binding protein 1 (YBX1), thus initiating FZD7 transcription and promoting intrahepatic cholangiocarcinoma progression. Wu et al.^95^ have reported that circYap halts the initiation of Yap translation in breast cancer cells by binding Yap mRNA, eIF4G, and PABP. CircPPP1R12A encodes a novel peptide, circPPP1R12A-73 aa, that promotes CRC progression by activating the Hippo-YAP signaling pathway^96^. In GC, Liu et al.^98^ have shown that YAP1 inhibits circRNA-000425 transcription, thereby promoting the oncogenic function of miR-17 and miR-106. Overall, this evidence suggests that circRNAs play roles in the function of the Hippo/YAP pathway function by acting as miR sponges or interacting with proteins, thus further influencing tumor-associated signaling pathways.

### circRNAs and the p53/Bcl-2 signaling pathway

**Figure 6:**
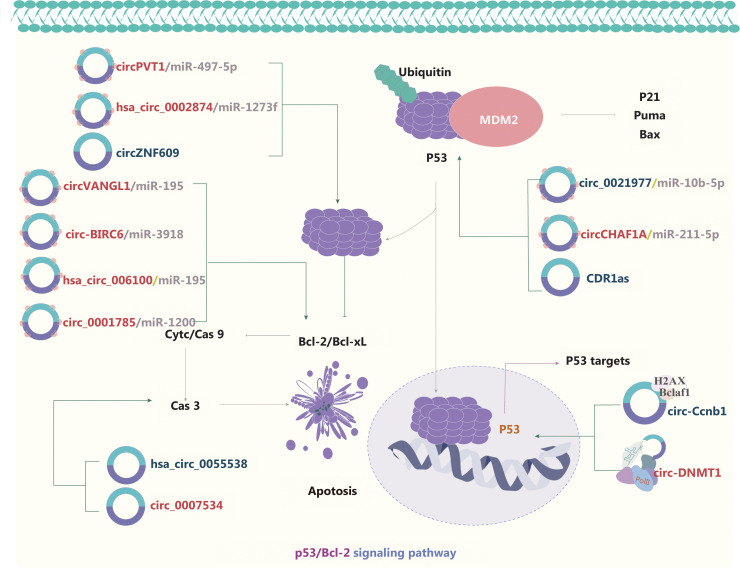
The molecular roles of circRNAs in the p53/Bcl-2 signaling pathway. *p53* is a common tumor suppressor gene that regulates the cell cycle and apoptosis through various pathways. A direct interaction between p53 and circRNAs preserves p53 by preventing its ubiquitination by E3 ubiquitin ligases (such as MDM2). circRNAs also affect the downstream targets of p53, such as Bcl-2 or Bcl-2/caspase, which mediates apoptosis and is involved in tumor development. Nine upregulated and 5 downregulated circRNAs are indicated; 7 circRNAs regulate the p53/Bcl-2 signaling pathway through ceRNA effects. Red: upregulated circRNAs; blue: downregulated circRNAs.

Tumor protein 53 (p53) participates in regulating the cell cycle and apoptosis through various pathways (**Figure 6**). In addition, the p53 signaling pathway is regulated by circRNAs, such as circVANGL1, circ-BRIC6, circ_0021977, circPVT1, hsa_circ_0002874, hsa_circ_006100, and circ-0001785, *via* the ceRNA mechanism^103–109^. Wild type p53 protein (wt-p53) is a homo-tetrameric transcription factor that serves as a tumor suppressor regulating the transcription of downstream target genes^110–112^. Mutant p53 (mut-p53) loses its tumor suppressive functions and gains tumor-promoting activities, known as gain-of-function (GOF) activities. Efficient mut-p53 GOF activity requires high mut-p53 protein expression levels in cancer cells. The formation of a hetero-tetrameric mut-p53/wt-p53 complex inhibits the tumor suppression function of the remaining wt-p53, thus leading to tumor cell proliferation, survival, migration, and invasion. Interestingly, crosstalk between mut-p53 and circRNA has been reported^113^. For example, circPVT1^106^ and circ-Ccnb1^114^ are activated by mut-p53. In a cohort of 115 patients with head and neck squamous cell carcinoma (HNSCC), circPVT1 and mut-p53 have been found to be over-expressed in tumor tissues compared with normal tissues. Mechanistically, the mut-p53/YAP/TEAD complex has been proposed to enhance circPVT1 transcription^106^. In breast cancer cells, wt-p53 enhances circ-Ccnb1 expression, whereas wt-p53 repression or mut-p53 expression suppresses circ-Ccnb1 expression. Circ-Ccnb1 dissociates the CyclinB1/Cdk1 mitotic complex, thereby suppressing cell invasion and tumorigenesis^114^. Mechanistically, a direct interaction between p53 and circRNAs has been speculated to prevent the destruction of p53, and this interaction may inhibit ubiquitination by E3 ubiquitin ligases (such as MDM2) and subsequent degradation of p53^113^. Binding of the circRNA CDR1as to p53 disrupts the p53/MDM2 complex, thus leading to p53 stabilization in glioblastoma^110^. In contrast, binding of circ-DNMT1 to p53 promotes the nuclear translocation of both p53 and AUF1, which in turn upregulates *DNMT1* expression and leads to the inhibition of *p53* expression in breast cancer^24^. circRNAs also affect Bcl-2, a downstream target of p53, and consequently mediate apoptosis in NSCLC, HCC CRC, and GC cancer cells^103–105, 108,115^. Moreover, hsacirc_0055538, circ_0001785, and circ_0007534 regulate the Bcl-2/caspase axis, and consequently induce or inhibit apoptosis in OSCC and osteosarcoma^109,112,116^. Notably, the formation of feedback loops is a novel mechanism of regulation in the p53 signaling pathway. In glioma, the FMR1/circCHAF1A/miR-211-5p/HOXC8 feedback loop regulates proliferation and tumorigenesis *via* MDM2-dependent p53 signaling^117^. Carcinogenesis is most frequently driven by *p53* mutations or inactivation, whereas p53/MDM2 complex formation controls p53 stability. Therefore, p53 has become one of most attractive therapeutic targets in cancer.

### circRNAs and the TGF-β/Smad signaling pathway inducing EMT

**Figure 7:**
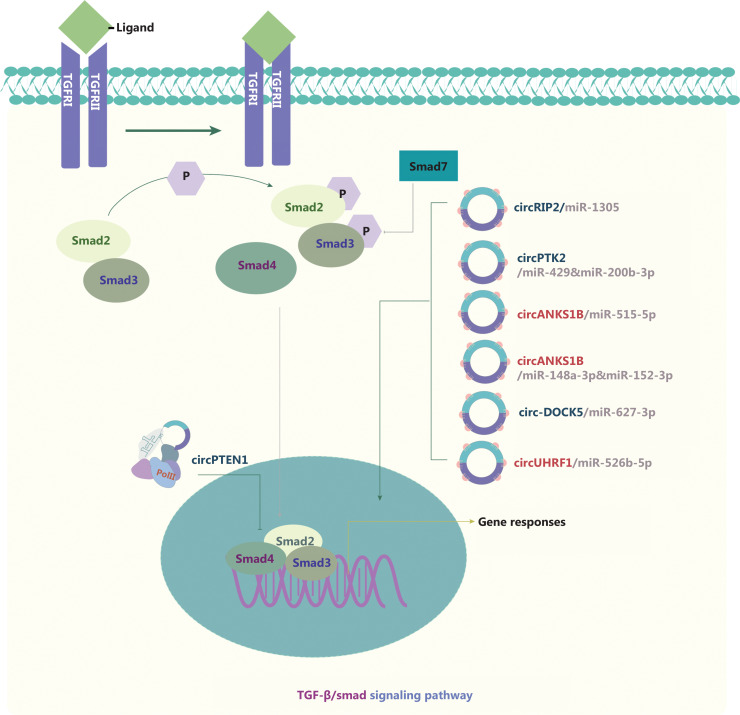
Molecular roles of circRNAs in the TGF-β/Smad signaling pathway. TGF-β signaling occurs *via* membrane-bound heteromeric serine-threonine kinase receptor complexes that are activated by TGF-β ligands and the subsequent phosphorylation of Smad family members. Smad members accumulate in the nucleus and act as transcription factors regulating target gene expression. Activation of canonical TGF-β signaling represses epithelial gene expression, and the resulting TGF-β-induced EMT has a central role in cancer metastasis. Three upregulated and 4 downregulated circRNAs are indicated; 4 circRNAs regulate the TGF-β/Smad pathway through ceRNA effects. Red: upregulated circRNAs; blue: downregulated circRNAs.

TGF-β1 canonical signaling is activated through receptor-regulated Smad (R-Smad), which elicits transcriptional responses by binding Smad binding elements in cell nuclei, thus repressing epithelial gene expression^118^. TGF-β1-induced EMT is an initiating and sustained step that plays a central role in cancer metastasis (**Figure 7**). circRNAs are also involved in the mechanism through which the TGF-β/Smad pathway positively regulates the growth and metastasis of various cancers. Wang et al.^119^ have demonstrated that circPTK2 is significantly downregulated in NSCLC cells and negatively correlates with TGF-β-induced EMT. In triple negative breast cancer (TNBC), circANKS1B interacts with miR-148a-3p and miR-152-3p, thus increasing the expression of upstream transcription factor 1 (USF1), which then binds TGF-β1^120^. Both circPTK2 and circANKS1B promote or suppress EMT in carcinogenesis *via* the ceRNA mechanism. CeRNA mechanism also applies to the function of circRIP2^121^ in bladder cancer and circANKS1B^122^ in OSCC. CircPTEN1 binds the MH2 domain of Smad4, disrupts its interaction with Smad2/3, and consequently suppresses the expression of its downstream genes associated with TGF-β-induced EMT^123^. In addition, the TGF-β/Smad signaling pathway has been found to be regulated by feedback loops formed by circRNAs such as circ-DOCK5^124^ and circUHRF1^125^.

Herein, we have focused on the canonical pathway associated with tumorigenesis and cancer progression. However, circRNAs also interact with other signaling pathways, such as the MAPK and JAK/STAT pathways in carcinogenesis^126^. Any aberrant interplay between such pathways can lead to breakthroughs in cancer progression. Together, these findings indicate that circRNAs have crucial roles in modulating the hallmarks of cancer cells.

## The landscape of circRNA databases

Initially, RNA-seq algorithms were inefficient in distinguishing back-splicing junctions from the corresponding circular structures. However, advances in high-throughput RNA sequencing have led to the rapid establishment of circRNA databases. These databases facilitate in-depth research to elucidate the biological functions of circRNAs as well as the underlying mechanisms. Increasing data resources specifically designed for circRNAs are now emerging. In this section, we discuss these databases and tools, which are classified into 3 groups: circRNA annotation databases, functional analysis databases, and human disease databases (**Table 2**).

**Table 2:** Content and web addresses of various circRNA databases

	Database	Short description	URL
Annotation information	circBank	Novel circRNA nomenclature system	http://www.circbank.cn
	circBase	circRNAs in humans, mice, *C. elegans*, and *D. melanogaster*	http://www.circbase.org/
	circAtlas	circRNAs in humans, macaques, mice, rats, pigs, chickens, and dogs	http://circatlas.biols.ac.cn/
	CIRCpedia V2	circRNAs in humans, mice, rats, zebrafish, flies, and worms	http://www.picb.ac.cn/rnomics/circpedia/
	deepBase v2.0	circRNAs across 19 species	http://biocenter.sysu.edu.cn/deepBase/
Function analysis tool	CircFunBase	circRNA-miRNA interaction networks	http://bis.zju.edu.cn/CircFunBase/
	circlncRNAnet	Coding-non-coding-expression network; RBP interactome	http://app.cgu.edu.tw/circlnc/
	Circlnteractome	Potential IRE, RBP, and miRNA binding sites on circRNAs	https://circinteractome.nia.nih.gov/
	TRcirc	Transcription factors; methylation levels; H3K27ac signals; super-enhancers	http://www.licpathway.net/TRCirc/view/index
	circRNADb	Protein-coding potential; relevant mass spectrometry information	http://reprod.njmu.edu.cn/cgi-bin/circrnadb/circRNADb.php
	CircCode	Identification of circRNA coding ability	https://github.com/PSSUN/CircCode
	CircPro	Identification of circRNA coding ability	http://bis.zju.edu.cn/CircPro
	CircNet	circRNA expression profiles, circRNA-miRNA sponge regulatory network	http://circnet.mbc.nctu.edu.tw/
	exoRBase	circRNA/lncRNA/mRNA in exosomes	http://www.exoRBase.org/
Human diseases	TSCD	Tissue-specific circRNAs in human and mouse tissues	http://gb.whu.edu.cn/TSCD
	circRNA-disease	circRNA-disease association; circRNA expression profile	http://cgga.org.cn:9091/circRNADisease/
	CircR2 Disease	Functions and molecular mechanisms of circRNAs in diseases	http://bioinformatics.zju.edu.cn/Circ2Disease/index.html
	CSCD	Cancer-specific circRNAs; ORFs; RBP-binding sites; miRNA target sites	http://gb.whu.edu.cn/CSCD
	MiOncoCirc	circRNAs from clinical cancer samples	https://mioncocirc.github.io/
	Circ2Traits	miRNA-circRNA-mRNA interaction networks with disease	http://gyanxet-beta.com/circdb/
	Circ2Disease	Manually curated database of experimentally validated circRNAs in human disease	http://bioinformatics.zju.edu.cn/Circ2Disease/index.html
	Circad	Comprehensive manually curated resource of circular RNA associated with diseases	http://clingen.igib.res.in/circad/

### Annotation databases

CircBank^127^, CircBase^128^, CircAtlas^129^, CIRCpedia v2^130^, and deepBase v2.0^131^ consolidate published circRNA articles and integrated RNA sequencing data. These tools provide comprehensive annotation information of circRNAs across species, including circRNA ID, genomic length, transcripts, genomic symbol, and relevant annotation. CircBank^127^ also collects data on circRNA conservation, circRNA protein-coding potential, m6A modification, miR-binding sites, and circRNA mutations. Furthermore, CircBank has established a novel nomenclature system based on the host gene name, start position, and end position. This system is based on the different search criteria used to obtain functional information for circRNAs. CircAtlas provides information on circRNA-miR interactions or circRNA-RBP interactions in 7 vertebrate species (humans, macaques, mice, rats, pigs, chickens, and dogs). CIRCpedia v2 provides circRNA annotations and expression features in various cell types and tissues related to 6 species^130^.

### Functional analyses databases

Evidence suggests that circRNAs play critical regulatory roles at the transcriptional and post-transcriptional levels by acting as miR/protein sponges, RBP-binding molecules, regulators of transcription, templates for translation, and components of exosomes^132^. Although most of their functional patterns remain undocumented, many databases that collect experimentally supported or putative circRNA-associated interactions are publicly available. CircFunBase is a web-accessible database providing a convenient visualized representation of circRNA-associated miRs/RBP interaction networks^133^ in the context of the genome. CirclncRNAnet is an integrated web-based resource that maps functional networks of long non-coding RNAs (lncRNAs) on the basis of uploaded NGS-based matrix data^134^. CircInteractome is a comprehensive knowledgebase that can be used to predict the potential RBP-binding sites of circRNAs, identify potential IRESs, and design siRNAs and primers^135^. CircInteractome predicts circRNA-RBP interactions on the basis of CLIP-seq data from starBase v2.0, whereas CircFunBase uses the RBP-circRNA interactions predicted directly by CircInteractome. TRCirc provides transcriptional regulatory information on circRNAs, including expression and methylation levels, H3K27ac signals in regulatory regions, and super-enhancers associated with circRNAs^136^. Although circRNAs were once classified as non-coding RNAs, the potential for circRNA translation is becoming increasingly clear. As such, circRNADb focuses on protein-coding annotations and provides genomic information, mass spectrometry evidence, and putative IRES and ORF sites^137^. CircCode is a Python 3-based framework that can be used to investigate the translational potential of circRNAs in humans and *Arabidopsis thaliana*^138^. CircPro can be used to predict the protein-coding potential of circRNAs and identify junction reads according to Ribo-seq data. CircNet provides information such as circRNA expression profiles, circRNA-miR sponge regulatory networks, and circRNA-gene-miR regulatory networks^139^. In addition, circRNAs may play roles in paracrine or endocrine regulation *via* exosomes^140^. ExoRBase contains details on circRNAs in human blood exosomes, and contributes to the study of exo-circRNAs and diseases^141^.

### Databases associated with human diseases

The application of databases for use in bioinformatics methods is important in identifying novel circRNA-disease associations. The systematic collection and management of circRNA-disease association data are critical for exploration of the clinical importance of circRNAs. The tissue-specific circRNA database (TSCD) contains information relating to the systematic analysis of tissue-specific circRNAs, and can be used to identify novel biomarkers of organogenesis and disease development^142^. The circR2Disease database provides a platform to investigate the pathological mechanisms of disease-associated circRNAs identified experimentally^143^. The cancer-specific circRNA database (CSCD) was constructed from RNA-seq datasets from tumor and normal tissue samples to serve as a resource for functional studies of cancer-specific circRNAs^144^. This database can be used to identify potential functions and predict candidate circRNAs with the potential for translation *via* metal responsive elements (MREs), RBPs, and ORFs. The MiOncoCirc database, which was established on the basis of exosome capture sequencing of clinical human cancer samples, provides comprehensive data including circRNAs from metastases, primary tumors, and very rare cancer types^145^. Circ2Traits focuses on the construction of circRNA-miR-mRNA networks, and is used to infer interactions between circRNAs and disease-associated miRs^146^. Circ2Disease contains 725 experimentally supported associations between 100 diseases and 661 circRNAs^147^. The Circad database is a collection of experimentally confirmed associations between circRNAs and diseases^148^. In addition, it contains circRNA annotation details, including the name, genome locus, and associated disease.

The rapid development of computational algorithms has facilitated the construction of novel computational models for the prediction of circRNA-disease associations, which can aid in the diagnosis and treatment of diseases^2^. For example, Locality-Constrained Linear Coding can be used to predict circRNAs associated with human diseases by integrating known circRNA-disease association, circRNA semantic similarity network, disease semantic similarity network, reconstructed circRNA similarity network, and reconstructed disease similarity network data^149^. Identification of circRNAs associated with diseases can contribute to a better understanding of the pathogenesis, diagnosis, and treatment of diseases.

## Conclusions and perspectives

In this review, we outlined current knowledge regarding the key roles of circRNAs in tumorigenesis. Complex circRNA regulatory networks have important implications in tumorigenesis and progression, as well as the development of novel treatments. circRNAs are enriched in the PI3K/AKT/mTOR, Wnt/β-catenin, Notch, Hippo, p53/Bcl-2, and TGF-β/Smad signaling pathways, and are abnormally expressed in different tumor types. Moreover, the interactions between circRNAs and signaling pathways show great potential for identifying novel therapeutic targets and diagnostic biomarkers^17,150^. Treatments based on nucleic acids represent a major breakthrough in the pharmaceutical field. Indeed, exogenous circRNAs can be used as miR sponges to prevent or enhance target mRNA expression, through placement of a series of MREs after a reporter gene. In addition, circular carriers have specialized secondary structures that form more durable and stable miR sponges than their linear counterparts. Furthermore, recent studies have shown that exogenous circRNAs promote therapeutic effects by activating the immune system^151,152^.

Collective biomedical databases and tools have been developed for deciphering circRNA-associated activities and their underlying mechanisms. Therefore, we focused on the circRNA-associated databases in this review. Each of these resources has unique aspects and strengths. However, the reliability and accuracy of their sources must be considered, given the variations in some results, possibly because of differences in experimental results, sample specificity, and the diversity of sequencing methods used to obtain the data. In addition, clear differences in sensitivity and precision exist among the various algorithms used in the different databases. Therefore, unified standards should be established to compensate for such differences and address these deficiencies. In addition, rapid advances in computational prediction algorithms have led to the generation of many computational models, such as scoring function-based models, which have been developed for the prediction of potential non-coding RNA-disease associations. Consequently, thousands of non-coding RNA-disease associations with non-coding RNAs, including circRNAs, lncRNAs, and miRs, have now been identified in eukaryotic organisms^149,153,154^. For example, Chen et al.^155^ have recently published the Neighborhood Constraint Matrix Completion for miR–Disease Association prediction model to predict potential miR–disease associations. According to Chen et al.^153^, analysis of available lncRNA–disease associations and prediction of potential human lncRNA–disease associations have become important bioinformatics projects. Comprehensive knowledge of non-coding RNA-disease associations would aid in understanding of human complex disease mechanisms; identification of disease biomarkers; and disease diagnosis, treatment, prognostication, and prevention.

The roles of circRNAs in mediating gene expression at the post-transcriptional level are a new focus of research on gene regulation in cancer. However, despite the rapid advances in the biological characterization of circRNAs, the mechanisms underlying their functions remain to be fully elucidated. For example, whereas circRNAs are now known to be retro-transcribed *in vivo*, and inserted back into the genome, thereby generating pseudogenes^156^, the underlying molecular mechanisms are unclear. Although molecular expression and function are often coupled and coordinated to some extent, the balance between circRNAs and their linear isoform transcripts, and the factors that dictate the dynamic generation and degradation rate is as yet undefined. The topological structures of circRNAs and their mechanisms of binding miRs/proteins are also unclear. Although some circRNAs can be translated into peptides, the underlying mechanism and the identity of peptide modulators is another area for future research. With the development of RNA sequencing techniques, improvements in databases, and continued research efforts in this area, some of these questions may be answered, and may ultimately enable the identification of novel cancer biomarkers and therapeutic targets.
